# Validation of an actuator line CFD model for tidal rotors in waves

**DOI:** 10.1016/j.jfluidstructs.2026.104583

**Published:** 2026-08

**Authors:** Federico Zilic de Arcos, James McNaughton, Christopher R. Vogel, Richard H․ J. Willden, Grégory Pinon

**Affiliations:** a Laboratoire Ondes et Milieux Complexes, LOMC-UMR 6295 CNRS, Université Le Havre Normandie, 53 Rue de Prony, 76600, Le Havre, 76600, Normandie, France; b Maritime Research Institute Netherlands, Haagsteeg 2, Wageningen, 6708 PM, Netherlands; c Department of Engineering Science, University of Oxford, Parks Road, Oxford, OX1 3PJ, Oxfordshire, UK

**Keywords:** Tidal rotor, Waves, Experiments, CFD, Volume of fluid, Actuator line model

## Abstract

This paper presents an analysis of two tidal rotors in close side-by-side proximity under homogeneous currents and surface waves. Multiphase Large Eddy Simulations with a Volume of Fluid free surface model, and an Actuator Line representation of the rotor are used to simulate their behaviour. The rotor was simulated in proximity to the free surface and to a vertical symmetry plane, mimicking twin-rotor towing tank experiments. Numerical and experimental results are compared, showing that the model accurately predicts loads and power at integrated and blade levels for a range of wave frequencies. Both model and experiments show that waves have a modest effect on mean thrust and power, but induce significant fluctuations. The predicted load fluctuations show a good agreement with experiments although with minor frequency-dependent amplitude underestimations and phase differences which suggest inertial effects not captured by the numerical model. The numerical results also show that waves affect wake development downstream, increasing mixing and driving the formation of large vortex structures dependent on wave frequency. Rotor induction, however, is observed to have a modest influence on the propagation of wave kinematics outside the wake region.

## 1 Introduction

Tidal rotors deployed at sea are likely to be affected by turbulence, shear, surface waves, and wave-induced motions in the case of floating devices. These can induce significant fluctuations in rotor thrust and power performance, which are likely to impact fatigue and the quality of power delivery (Adcock et al., 2021).

Surface waves have the potential to be the largest driver of load and power fluctuations on tidal rotors (Scarlett and Viola, 2020). Several experimental studies have reported substantial thrust and power fluctuations due to wave action: Galloway et al. (2014) reported fluctuations in the median flapwise root bending moment on a blade of up to 175%; while Draycott et al. (2019) reported fluctuations about the mean between 7% and 65% for integrated thrust, and 13% to 165% for power. Several other studies report load and power fluctuations of the same order of magnitude (see, e.g., Barltrop et al., 2007, Faudot and Dahlhaug, 2012, Gaurier et al., 2013, Guo et al., 2018, Dufour et al., 2022, or Watanabe et al., 2023). Most of these studies also indicate that time-averaged loads are largely unaffected by the combination of waves and homogeneous currents. The magnitude of the effects of surface waves on tidal rotors, however, is strongly case-dependent and is influenced by factors such as wave amplitude, frequency, incidence angle, and the rotor’s submergence depth, as waves alter the flow profile that the turbine is exposed to (Guo et al., 2018, Kolekar et al., 2019). Rotor design, control strategy, and the response of other subsystems such as floating platforms and moorings are also likely to influence the magnitude of dynamic loads on rotor blades (Zilic de Arcos et al., 2023a).

Wave-driven load fluctuations on tidal turbines are caused mainly by temporal and spatial velocity variations across the water column. The spatial component is associated with wave kinematics decaying with depth and wave yaw angle. Even in a steady sheared profile, turbine blades encounter different flow speeds as they rotate, inducing time-dependent load fluctuations in rotational and blade frequencies (Draycott et al., 2020). Wave-induced velocities, however, also fluctuate in time at the wave frequency, driving further complexity in blade loads.

Tidal turbines are also affected by blockage, defined as the interaction between the rotor and the surrounding boundaries of a fluid domain, which affects rotor loads and power performance. The simplest form of blockage is the isotropic interaction between a rotor and the boundaries constraining the domain (Glauert, 1947). The effects of isotropic blockage are normally seen in the form of an increase in maximum thrust and power on tidal turbines caused by changes in static pressure gradient and an increased mass flux through the rotor (Zilic de Arcos et al., 2020a). Tidal rotors are likely to be deployed close to channel boundaries and in array configurations. Multi-rotor interactions (McNaughton et al., 2023, Nishino and Willden, 2012) and non-isotropic interactions with the free-surface (Kolekar et al., 2019) affect rotor loads and power while inducing non-uniform flow fields that further affect dynamic rotor loads, power, and wake development (Li et al., 2021). The combination of non-isotropic blockage and waves creates a complex environment for the operation of tidal turbines. Further research is required to understand the impact of these dynamic phenomena on tidal turbines and to determine and validate adequate modelling techniques (Adcock et al., 2021).

The effects of waves and non-isotropic interactions between the rotor and the free surface are discussed in the present work. Our paper presents the case of a tidal rotor near the free surface and a vertical symmetry plane to model twin-rotor operation. The rotor is simulated under a steady homogeneous current and regular waves, reproducing the experiments of McNaughton et al. (2025). To simulate the experiments, we used an Actuator Line Model (ALM) representation of the rotor embedded within a multiphase Large Eddy Simulation (LES) Computational Fluid Dynamics (CFD) model. The numerical results are compared to experiments, highlighting the strengths and limitations of the model predicting steady and deterministic dynamic loads under a space and time varying flow field. The unrestricted access to the rotor model and the flow field is then used to interpret the experimental results, to explore wave effects on the rotor wake and the phase of wave kinematics, and to assess the limitations of the numerical model through experimental-numerical comparisons.

The manuscript is organised as follows. First, a description of the ALM implementation is presented, which includes the implementation of a new sampling method (Zormpa et al., 2025) and the use of lift and drag coefficients extracted from blade resolved RANS-CFD simulations. This is followed by a verification and validation of the numerical model through comparisons with blade resolved steady CFD simulations and the experimental results of McNaughton et al. (2025); a continuation of the discussion on the wave and azimuth phases of load fluctuations, previously introduced in the experimental paper (McNaughton et al., 2025) and now extended using the CFD results; and finally concludes with a discussion on wave effects on the turbine wake and the impact of rotor induction on the phases of wave kinematics.

## 2 Methods

### 2.1 Turbine description

This study is based on the 1.2-metre diameter 3-bladed tidal turbine designed by Cao (2020). The blade is composed of a modified FX-84-W-140 hydrofoil, projected radially, with a blunt trailing edge obtained through the application of a uniform thickening function over the trailing half of the foil. The foil’s maximum efficiency is found at an angle of attack of approximately 6^∘^, while the rotor’s peak performance is obtained at a tip-speed ratio (TSR) of approximately $\lambda =\omega R/U_{\infty }=6.2$, where *ω* is the turbine rotational speed, *R* the rotor radius, and *U*_∞_ the undisturbed flow speed.

**Fig. 1:**
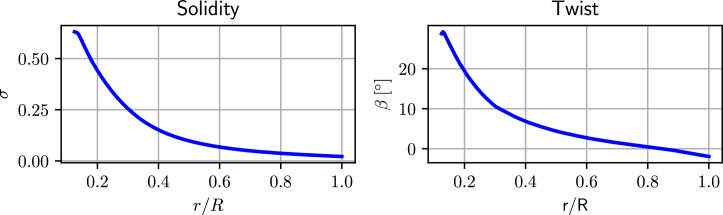
Spanwise distributions of blade solidity and twist angle.

The rotor was developed using a design and optimisation method based on a RANS CFD model with an Actuator Disc (AD) rotor representation. This AD model relies on blade-element theory to determine forces based on local flow conditions (Schluntz and Willden, 2015). The optimisation model considers local blockage and constructive interference effects (Nishino and Willden, 2012) as part of the blade optimisation process. The blockage-optimised rotor showed an increased blade solidity and reduced variation in twist along the blade, enabling an increased power conversion compared to a rotor designed for unblocked flow (Wimshurst and Willden, 2016). Spanwise distributions of twist, *β*, and solidity, σ=cN/2πR, with *c* the local chord and *N* number of blades, are shown in Fig. 1.

McNaughton et al., examined and validated the blockage and multi-rotor interactions considered to design this rotor in flume (McNaughton et al., 2022) and towing tank experiments (McNaughton et al., 2023). These studies demonstrated constructive interference effects, with turbine and fence-averaged power uplifts of up to 10.8% obtained (having accounted for global blockage effects) through multi-rotor interactions.

### 2.2 Experimental campaign

The numerical study follows the experimental campaigns performed at QinetiQ’s Haslar towing tank. The tank has a cross section of 12.2 metres width, 5.4 metres depth, and a usable section of approximately 150 m in length. Two 1.2 m diameter turbines of identical design and rotational direction, previously tested at FloWave (McNaughton et al., 2022) and SSPA (McNaughton et al., 2023), were fixed vertically to the towing carriage, with their hub centrelines positioned 1 m below the free surface and with a tip-to-surface clearance of 0.4 m (2*R*/3). Turbine lateral position was symmetrical about the tank centre-plane, with an inter-turbine spacing of 0.3 m (*R*/2).

Tests were performed for different sets of rotational speeds and regular head waves of different amplitudes and wave frequencies. Throughout the different tests, both rotors were configured to have identical rotational speeds and direction, while the carriage speed was maintained constant at $U_{\infty }=1.0\;$ m/s. Note that a previous towing tank campaign at SSPA had already demonstrated these rotors behaved with Reynolds number independence at towing speeds above 0.9 m/s (McNaughton et al., 2023).

Measured quantities in each turbine included rotational position and speed, integrated thrust and torque, and root bending moments in flapwise and edgewise directions. Six wave gauges were installed in two different layouts. Twenty seconds of zeroing measurements were captured before each trial to quantify and reduce sensor drift, followed by approximately 140 s of testing time. A settling time of 15 minutes was used between tests. The usable length of the data captured on each trial varied across tests and conditions. Data was processed to select measuring windows free from the initial transients and wave reflections, with lengths between 20 and 40 seconds of usable data per test. Further details on the experiments, testing conditions, data processing techniques, and equipment are presented in McNaughton et al. (2025).

### 2.3 Case selection

The experimental campaign consisted of runs with and without surface waves, and at different rotational speeds. Two subsets of the experiments at QinetiQ were selected for this numerical study: a *λ*-varying subset without waves (*λ* ∈ 5.5, 6.0, 6.2, 6.7, 7.2, 7.6, 8.6), and a subset with different wave frequencies and a target wave amplitude of 0.1 m, with the rotors operating at constant λ=6.2.

Wave conditions are referenced by their nominal frequency which corresponds to the frequency of the wave generator. Table 1 shows a summary of the wave frequencies in this subset, alongside the corresponding encounter frequencies relative to the moving towing carriage, and the calculated wavelengths.

**Table 1:** Summary of the simulated wave conditions, as well as correspondence between nominal wave frequencies, encounter frequencies relative to the rotor, and wavelengths.

Nominal frequency	Encounter frequency	Wavelength
*f* [Hz]	*f_e_* [Hz]	*L* [m]
0.40	0.50	9.76
0.50	0.66	6.25
0.60	0.83	4.34
0.65	0.92	3.70

### 2.4 CFD model

#### 2.4.1 Model description

We used a Volume of Fluid (VoF) multiphase CFD model with the isoAdvector scheme of Roenby et al. (2016) for interface advection and a PISO algorithm to simulate the towing tank where the experiments were performed. Simulations were performed using OpenFOAM v2012. A LES turbulence closure was used with the Wall-Adapting Local Eddy-Viscosity (WALE) subgrid scale model (SGS) of Nicoud and Ducros (1999) modified to act only on the water phase of the domain. This was done by masking the eddy viscosity using the volume fraction of water. This minor modification avoids non-physical perturbations to the free surface observed with the standard SGS while maintaining a good modelling accuracy in terms of surface elevation and wave kinematics, as shown in Section 2.4.3.

Note that in previous work (Zilic de Arcos et al., 2023b, Zilic de Arcos et al., 2025) we presented similar simulations where the stabilised k-*ω* SST turbulence model of Larsen and Fuhrman (2018) was used to reduce the wave dissipation associated with RANS turbulence modelling. This modified k-*ω* SST closure introduces a limiter to the specific dissipation rate *ω* to limit the unbounded production of turbulent kinetic energy predicted by RANS models in quasi-potential flows (Larsen and Fuhrman, 2018). Whilst this turbulence closure model is effective, our practical experience showed numerical stability issues when combined with the ALM and the isoAdvector scheme that were not seen with LES. Thus, we favour the latter option despite observing a negligible impact in the modelling of wave kinematics and ALM load predictions compared to previous simulations (Zilic de Arcos et al., 2025).

**Fig. 2:**
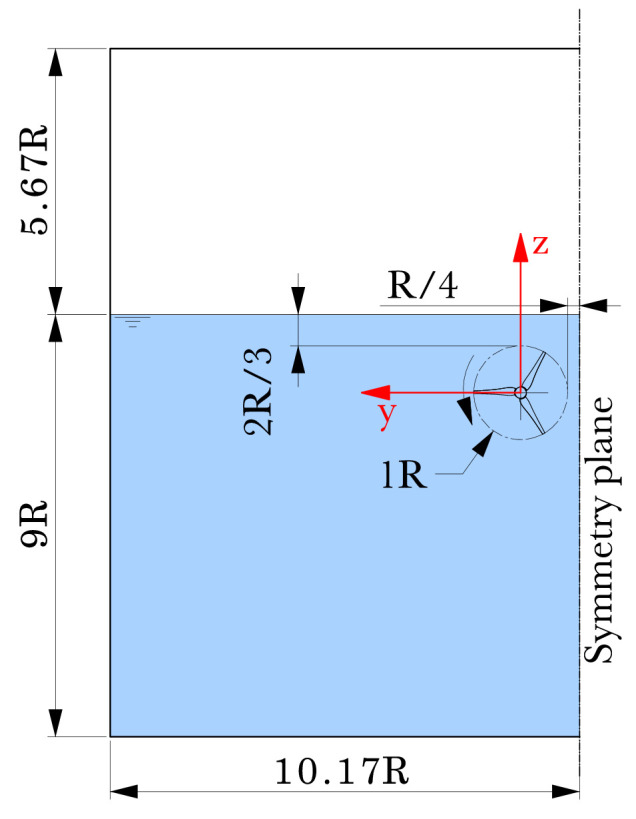
Diagram of the cross section of the simulated domain. The right-handed reference frame marks the origin of coordinates and positive lateral (*y*) and vertical (*z*) directions. Current and wave propagation directions (into the page) are aligned with the positive *x* axis. The origin is located at the rotor centre.

To reduce computational cost, the fluid domain was simulated using a symmetry boundary condition at the tank’s centre plane, with up- and downstream distances from the rotor plane of 10 and 26 Radii, respectively, and a cross section that follows the tank dimension. The cross section of the tank is shown in Fig. 2 alongside reference frame definitions. The use of a symmetry plane mimics the experimental multi-rotor operation with an inter-turbine (tip-to-tip) spacing of *R*/2 whilst allowing only one rotor to be simulated. The simulated rotors are contra-rotating. This is a difference with the experimental setup, although no significant impact on the results of the simulated rotor is expected. The Generating-Absorbing Boundary Condition of Borsboom and Jacobsen (2021) was used at the inlet and outlet of the tank to generate and absorb waves, respectively, as implemented in the waves2Foam library (Jacobsen et al., 2012), corresponding to prescribed velocity and volume fraction at the inlet, and a pressure outlet condition, respectively. No turbulence was forced in the domain due to the tests being conducted in a still water tank with negligible turbulence intensity levels (Willden et al., 2023). Waves and current conditions were prescribed using the stream wave theory of Fenton (1988), a non-linear model that describes wave kinematics relative to a moving reference frame, making it suitable to simulate current and waves. The rest of the boundaries are set as atmospheric at the top of the domain, and free slip for the bottom and the wall opposite to the symmetry plane.

**Fig. 3:**
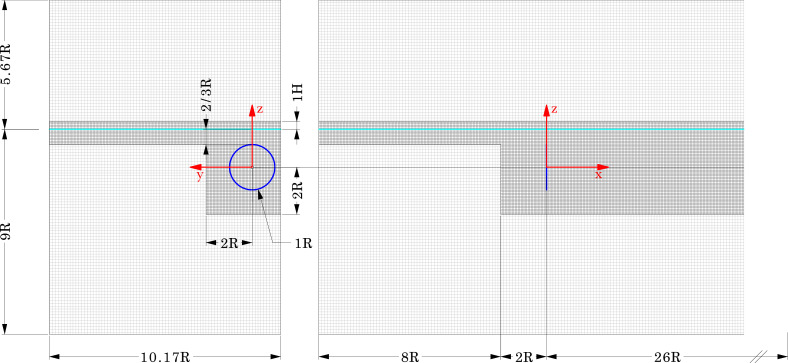
Diagram of the mesh domain and refinement zones. The darker zones show areas with a one-step mesh refinement. The origin is located at the rotor centre. Grid size not to scale.

The fluid domain was discretised using an isotropic hexahedral mesh, with a background size of 0.04 m and a one-level refinement zone for the free surface, rotor, and wake with a mesh size of Δx= 0.02 m. The refined region covers the entire free surface, extending vertically one wave height above the mean water level and two wave heights below (Windt et al., 2019), and a box region that spans from 2*R* upstream from the rotor plane to the outlet, and 2*R* to the bottom and side boundaries measured from the rotor’s centre of rotation. The mesh size in the refined zone had a resolution of 10 cells per wave height, a minimum of 185 cells per the shortest analysed wavelength, and 60 cells per rotor diameter. The mesh had a total 33.3 million cells. A diagram of the mesh domain and refinement zones is shown in Fig. 3.

#### 2.4.2 Actuator line method

The rotor was modelled using an Actuator Line CFD approach (Sørensen and Shen, 2002). Each blade is modelled through 50 collocation points aligned over the blade span using a cosine distribution to better resolve root and tip regions. Forces are introduced into the flow through sources in the momentum equations, and smeared onto the discretised domain using isotropic Gaussian distributions centred around each collocation point, with a constant core size ϵ=Δx and a smear radius of $R_{S}=3\epsilon$ (Troldborg, 2009). The forces in this model are determined using blade-element theory, with angle of attack and inflow speed determined using the line average method of Jost et al. (2018). This is a robust sampling method used to determine the local angle of attack of the flow approaching a three-dimensional blade, and was introduced in Zormpa et al. (2025) as the flow sampling technique for our Actuator Line implementation with a sampling radius of 1.1*R_S_*. The choice of the sampling radius is mostly arbitrary since calculated angles of attack are relatively insensitive to this parameter for diameters up to a few chords (Jost et al., 2018, Zilic de Arcos et al., 2020b).

The use of the line average sampling method reduces the dependency of thrust and power predictions on spatial and temporal discretisations, allowing for increased robustness and lower computational expense. Zormpa et al. also demonstrate an increased accuracy of their method’s power and thrust predictions by reducing the underestimation of blades’ axial induction in the flow, a phenomenon that has been attributed to viscous effects on the vortex core generated by the Actuator Line and shed vorticity (Meyer Forsting et al., 2019). While time-consuming corrections have been proposed to match lifting line theory (see, e.g., Meyer Forsting et al., 2019), this implementation of the line average method circumvents the issue by sampling the flow outside the Gaussian region where forces are applied, increasing the accuracy and robustness of the tool (Zormpa et al., 2025).

Lift and drag coefficients, used to determine sectional forces in the ALM, were extracted from three-dimensional blade resolved steady flow CFD simulations of the rotor. Extracting the lift and drag coefficients at different blade sections from blade resolved simulations allows to implicitly capture changes in polar coefficients that occur in proximity to the blade hub and tip, and that arise through hard-to-capture three-dimensional effects (Wimshurst and Willden, 2017b). This removes the need for empirical performance corrections such as, e.g., those proposed by Shen et al. (2005), and Wimshurst and Willden (2017a), which are required when using polar coefficients derived from two-dimensional foil experiments or simulations.

The blade resolved simulations used to produce the polar coefficients were conducted under simplified assumptions. One turbine blade in a third of a cylindrical domain was modelled using the same global blockage, inflow speed, and turbulence conditions as for the tank experiments and Actuator Line simulations. We used a single-phase steady-state model for these precursor simulations, with the Multiple Reference Frame (MRF) approach of Luo et al. (1994) to capture blade rotation in steady state, with a wall-resolved structured block-mesh ($y^{+}<1$ and a total cell count of 26 million for the one-blade domain), and a k-*ω* SST turbulence model (Menter et al., 2003). Thorough descriptions of the modelling strategy are presented by Wimshurst and Willden (2016) and Zilic de Arcos (2021); with validation in Wimshurst and Willden (2017b) and Willden et al. (2023).

**Fig. 4:**
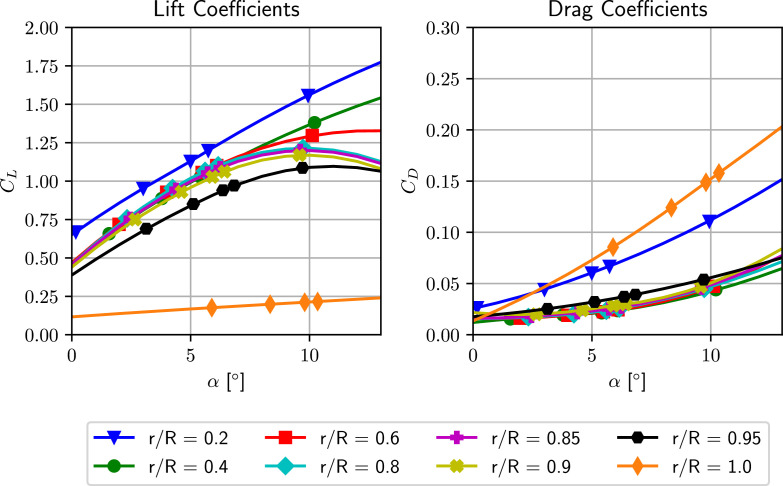
Lift and drag coefficients extracted from three-dimensional steady flow blade resolved simulations at different spanwise locations. The markers indicate data points extracted from simulations.

Five blade resolved simulations were conducted with tip-speed ratios *λ* ∈ [5.03, 6.20, 6.41, 7.04, 8.55]. Spanwise force distributions from the blade resolved simulations were combined with local angles of attack to calculate the lift and drag coefficients, *C_L_* and *C_D_*, required as inputs to the Actuator Line simulations. The lift and drag coefficients as functions of angle of attack *α* and local radius *r* are defined as:

(1) $$C_{L}\left(\alpha ,r\right)=\frac{L}{\frac{1}{2}\rho W^{2}c}$$

(2) $$C_{D}\left(\alpha ,r\right)=\frac{D}{\frac{1}{2}\rho W^{2}c}$$

with *L* and *D* the local lift and drag forces, *ρ* the fluid density, and *W* the relative inflow speed to the blade section (considers local blade rotation as well as axial and tangential inductions). Blade forces are computed integrating pressure and shear stress at different spanwise locations, and are projected onto a reference frame defined by the local inflow angle to determine the spanwise distributions of lift and drag. Inflow speeds, inflow angles and angles of attack are determined using the line average method with a sampling radius set to 1.1 chords (Jost et al., 2018). Each blade resolved simulation at a given TSR produces a single value of *C_L_* and *C_D_* at local *α* for each spanwise location (Zilic de Arcos et al., 2020b). The resulting lift and drag coefficients used with the ALM simulations are presented in Fig. 4.

The work of Zormpa et al. (2025) shows that the ALM combined with the line average sampling has a limited sensitivity to spatial and temporal discretisations. The ALM method, however, is still sensitive to the size of the force Gaussian defined by ϵ. In general, larger ϵ values favour lower force densities in the domain which lead to lower axial induction factors and higher angles of attack. The lower bound for ϵ is defined by the grid; the spatial resolution needs to be sufficient to resolve the vortex induced by the actuator line.

**Fig. 5:**
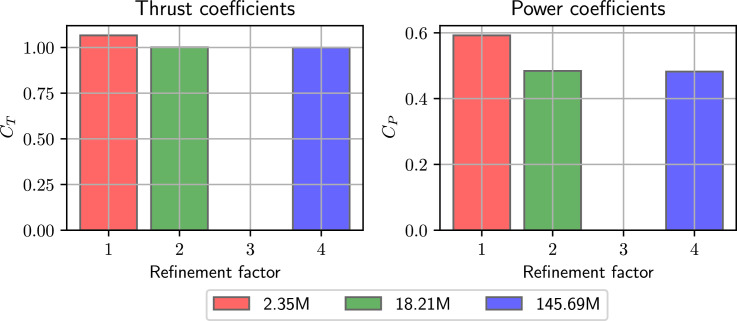
Thrust and power coefficients for the rotor operating at design tip-speed ratio with different mesh refinement factors. Coloured legends display the cell count for each case.

We evaluated the impact of grid resolution on the ALM by simulating the rotor in a single-phase domain with an isotropic global blockage equivalent to the channel simulations. In this case the turbine is placed at the centre of the domain far from boundaries or free surface. The rotor is simulated at design TSR and with three levels of mesh refinement. The power and thrust coefficient results are shown in Fig. 5.

The refinement factor of 2 in the figure is equivalent in spatial resolution to the open channel simulations, with the same isotropic hexahedral mesh configuration with background size of 0.04 m and a one-level refinement zone for the rotor and wake with a mesh size of Δx= 0.02 m. Note that the free surface refinement zone is omitted to reduce computational cost and, thus, enable the fine mesh simulation. The refinement factors of 1 and 4 correspond to the coarse and fine grids, respectively. The refinement is isotropic, with each cell divided by 2 in every dimension on each refinement step, increasing cell count by a factor of 8. The number of cells per diameter is of 30, 60 and 120 for the coarse, base, and fine grids, respectively. The results in the figure show negligible differences between the base and fine meshes (i.e., despite the cell count, the actuator line is sufficiently resolved in both cases), while the coarse grid suffers from an unresolved actuator line.

**Fig. 6:**
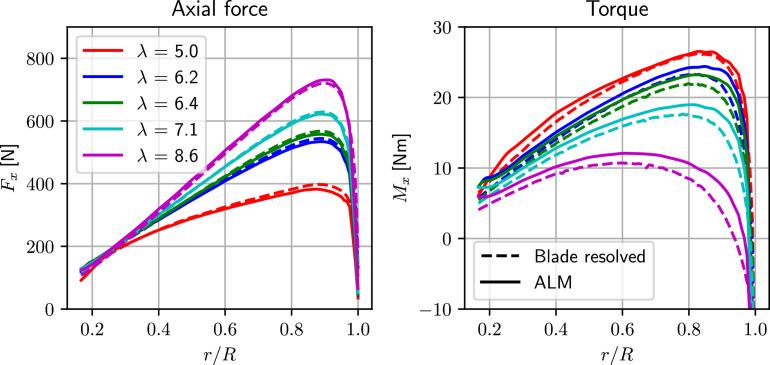
Thrust and torque distributions comparing ALM and blade resolved MRF models at different tip-speed ratios.

Using the base grid configuration with 60 cells per diameter across the rotor plane, a comparison between the spanwise thrust and torque distributions predicted by the blade resolved MRF and the ALM, simulated under equivalent domain, blockage and boundary conditions, is shown in Fig. 6. The results show a good agreement between both models in thrust and torque, although with some discrepancies that increase with TSR. Thrust, specifically, shows a better agreement between models than torque across the entire span of the blade.

**Fig. 7:**
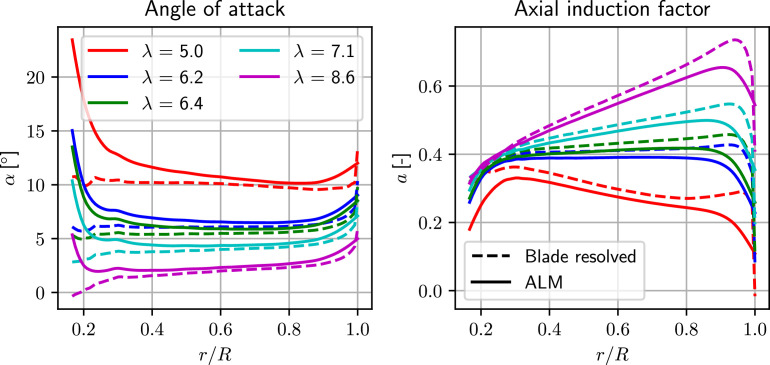
Angle of attack and axial induction distributions comparing ALM and blade resolved MRF models at different tip-speed ratios.

Angle of attack and axial induction factor distributions, $a=1-U_{X}/U_{\infty }$, with *U_X_* the local flow speed in the axial direction at the blade location, are shown in Fig. 7. These plots show similar results predicted by both models, with the largest discrepancies near the root region. Differences in this region are associated with the lack of a nacelle in the ALM and occur in a region of the blade where the production of thrust and torque is low, thus having a limited impact on the integrated results.

The differences in thrust and torque distributions can be traced to local flow vectors. Angle of attack differences between models are similar in magnitude at different TSRs over the region of the blade that contributes most to thrust and power production (40-85% of span). The difference as a proportion of local angle of attack, however, varies with TSR. The highest TSR, thus, shows the largest relative difference between models, which explains the reduced accuracy especially with respect to torque predictions in Fig. 6.

The plots also show that the ALM generally underestimates axial induction factors. As discussed before, the ALM is a representation of the forces generated by the rotor through a source term in the momentum equations without imposing a non-penetration condition. The lack of a non-penetration condition leads to a mass flux through the Actuator Line that does not exist in the case of a solid blade. The through-blade flux is a function of force density around collocation points. Reducing the size of the force Gaussian distribution would lead to an increased force density and, thus, higher axial induction factors, which in this case would tend to bring the Actuator Line results closer to blade resolved. The lack of a nacelle in the ALM also affects the results, though the impact of this simplification is limited to the root region as evidenced in the force, *α* and *a* distributions.

While a further increase in axial induction and, thus, a reduction in angle of attack error are possible, the force redistribution Gaussian cannot be smaller than the grid, as shown previously, and mesh resolution is limited by computational cost. The balance between accuracy and practicality led to the presented Actuator Line CFD model, which is considered a sufficiently robust and accurate method for the purpose of this study.

#### 2.4.3 Free surface modelling assessment

**Fig. 8:**
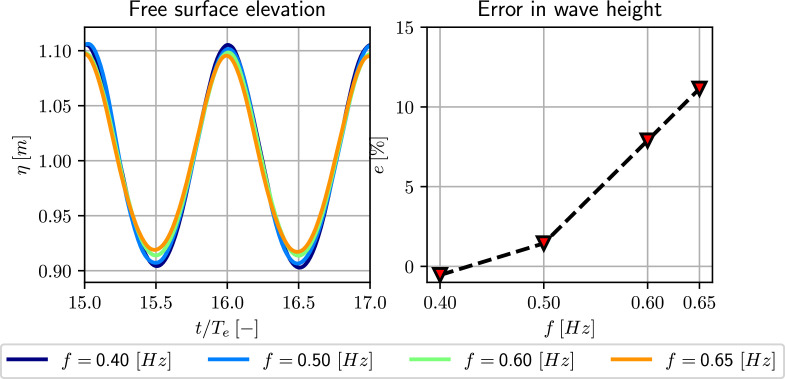
Simulated wave elevation at the rotor plane and relative error between target and modelled wave heights. Time is normalised by the encounter period $T_{e}=1/f_{e}$.

Fig. 8 shows computed surface elevation *η* at the rotor plane without the rotor. The figure also shows a quantification of the relative error *e* between the wave height obtained from the simulations and the target value. The results show varying errors that increase monotonically with frequency, ranging from approximately 0% for the wave with a frequency of f=0.40 Hz to 11% for the f=0.65 Hz case. The error variations between different cases are attributed to an increasing steepness with frequency (Ransley, 2015), as target amplitude is kept constant for different wave frequencies, as well as to the reduced number of cells per wavelength in the direction of wave propagation: the longest wave, f=0.40 Hz, is resolved with 488 cells per wavelength, while the shortest one, f=0.65Hz, by only 185 cells. Considering the variability in wave elevation in physical experiments reported by McNaughton et al. (2025), the simulated wave environment is considered adequate. Further mesh refinements could reduce wave dissipation, but at a non-practical increase in computational cost.

**Fig. 9:**
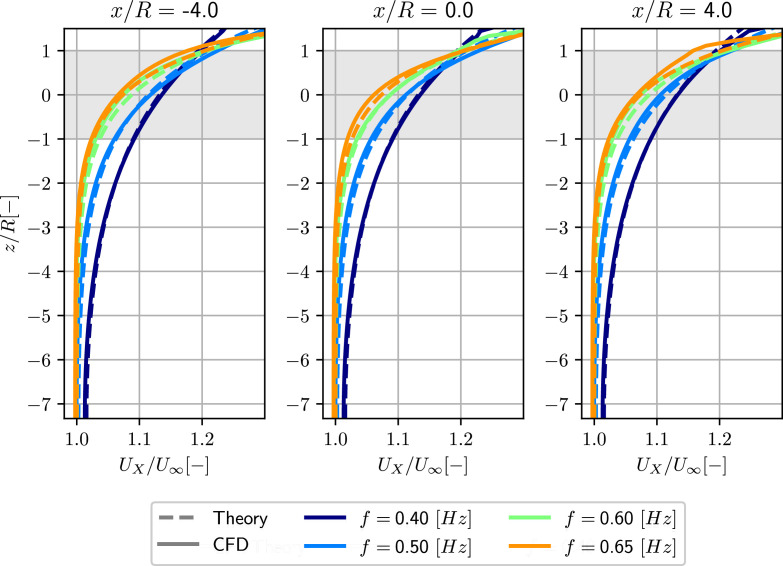
Comparison of CFD simulations without the rotor and the theoretical maximum horizontal velocity profiles at different axial locations. The shaded area marks the rotor region.

Fig. 9 shows profiles of maximum horizontal velocity (including wave-induced velocity) as a function of depth for the different wave cases. The plots compare the maximum horizontal velocity between the CFD and theoretical profiles at three different streamwise locations. Similar to the wave height analysis, these plots show a good agreement between theoretical and modelled results, with longer waves showing a better agreement and small relative differences in maximum flow speeds of -0.24% to 1.24% at the rotor centre, for the longest and shortest waves, respectively.

## 3 Results and discussion

### 3.1 Steady-state loads

**Fig. 10:**
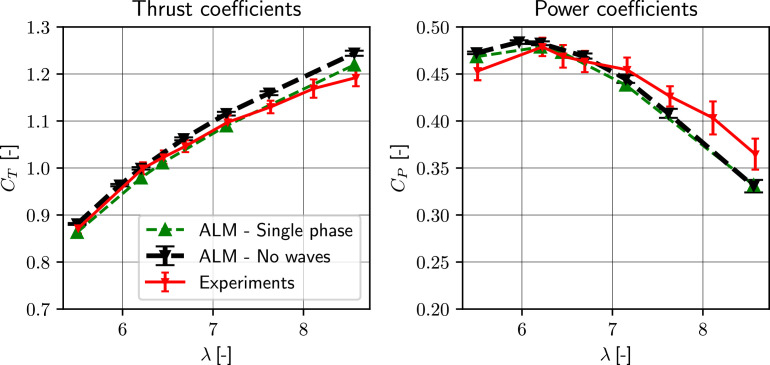
Integrated thrust and power coefficients, *C_T_* and *C_P_*, under steady flow conditions. The plots compare single-phase isotropic blockage and multiphase channel blockage Actuator Line CFD models with towing tank experiments.

Fig. 10 shows power and thrust coefficients, *C_P_* and *C_T_*, for the range of analysed tip-speed ratios. The figure shows a comparison between the single-phase isotropic blockage model used previously in the comparison with blade resolved simulations, the ALM simulations with the VoF approach that includes proximity to the free surface and symmetry boundary (referred to as channel blockage), and the experimental results for the turbines tested at QinetiQ. The error bars mark two standard deviations from mean values to highlight time-dependent fluctuations.

The comparison between the single-phase and VoF Actuator Line simulations shows a mild increase in thrust caused by the constructive interference and local blockage effects, as well as an increase in maximum power coefficient at tip-speed ratios close and below the design condition *λ* ≈ 6.2.

The ALM-VoF model shows a good steady-state agreement with the experiments, with relative errors from 0.3% and 0.8% of mean thrust and power near design TSR, to maximum differences of 4.2% and 9.8% on thrust and power, respectively, at the highest TSR.

### 3.2 Rotor loads and power performance in waves

**Fig. 11:**
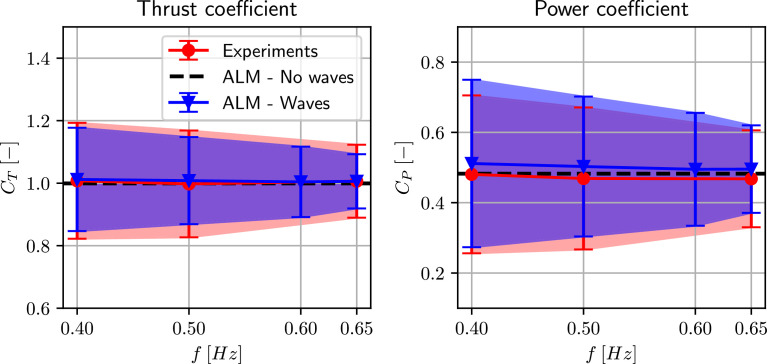
Thrust and power coefficients, normalised using mean flow speed, under different wave conditions of target amplitude 0.1 m and with the rotor operating at a constant rotational speed (λ=6.2). Markers indicate time-averaged results, while vertical bars and shaded areas represent two standard deviations from mean thrust and power for the cases with waves.

Fig. 11 shows whole-rotor thrust and power coefficients as a function of wave frequency, with the rotor operating at the design tip-speed ratio. The plots show time-averaged thrust and power, as well as thrust and power fluctuations represented by two standard deviations from the mean. The figure also compares the ALM results, with and without waves, with the experiments.

The *C_T_* and *C_P_* plots show, in agreement with literature, modest variations on time-averaged values when comparing the cases with and without waves. The mean ALM results also show a good agreement with the experiments for both power and thrust, with relative errors in the range of -1.6% to 1.3% for mean thrust, and 1.0% to 8.7% for power.

The thrust and power fluctuations induced by waves decrease with excitation frequency, as shown by the experimental and numerical results. This is attributed to the stronger wave kinematics induced by longer waves, as shown in Fig. 10, as well as to the faster decay of wave-induced velocities with depth observed for the shorter waves. Comparing the experimental and numerical results we observe that, while there is a good qualitative agreement for all cases, the relative difference between the fluctuation amplitudes decreases from 3.7% and 13.2%, for power and thrust at f=0.40Hz, to 15.3% and 28.9% for f=0.65Hz. This decrease in accuracy of simulated thrust and power fluctuations with frequency is attributed mainly to the underprediction of wave height and wave kinematics discussed in Section 2.4.3, although it seems likely that foil-scale dynamics (notably related to the use of steady lift and drag coefficients), as well as other phenomena such as added-mass forces and returning wake effects that are not adequately captured by the ALM, could also have an adverse effect on the model’s predictive capacity (Smyth, 2020, Smyth et al., 2023).

**Fig. 12:**
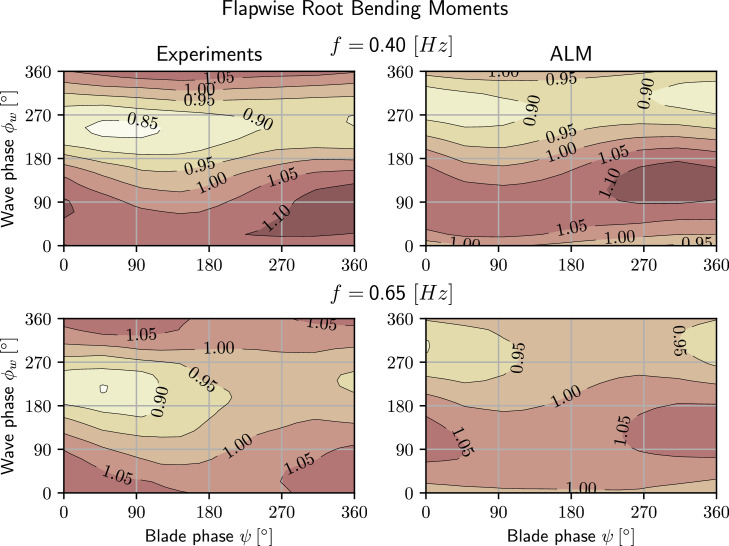
Comparison of flapwise root bending moments as a function of blade azimuth position, *ψ*, measured from top-dead centre, and wave phase, *ϕ_w_*, measured from the zero-surface upward crossing. The left column corresponds to the experimental data and the right column to the ALM simulations; the upper row corresponds to the f=0.40Hz and the lower to f=0.65Hz cases. Target wave amplitude is 0.1 m. Bending moments at each bin are normalised by wave and blade phase averaged RBMs.

**Fig. 13:**
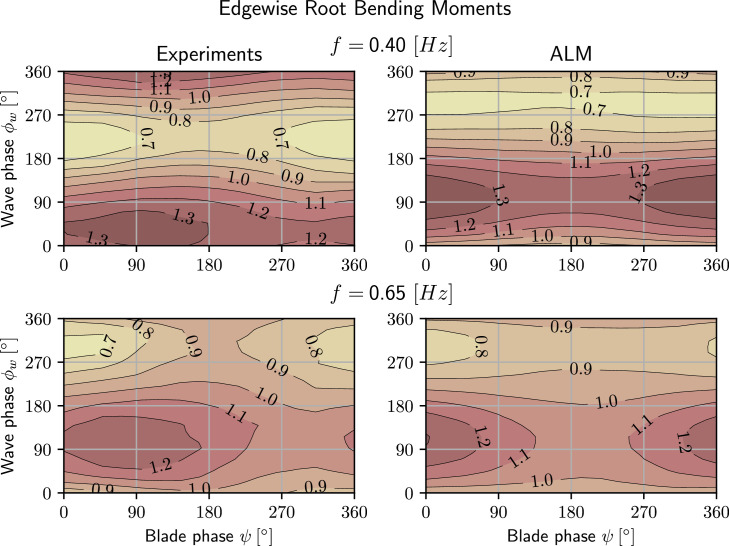
Comparison of edgewise root bending moments as a function of blade azimuth position, *ψ*, measured from top-dead centre, and wave phase, *ϕ_w_*, measured from the zero-surface upward crossing. The left column corresponds to the experimental data and the right column to the ALM simulations; the upper row corresponds to f=0.40Hz and the lower to the f=0.65Hz case. Target wave amplitude is 0.1 m. Bending moments at each bin are normalised by wave and blade phase averaged RBMs.

While whole-rotor power and thrust in regular waves can be approximated by a single harmonic (Guo et al., 2018, McNaughton et al., 2025), individual blade loads are more complex due to the contribution of inflow fluctuations at wave and blade frequencies, as well as due to the non-linear depth-dependent amplitude of vertical and horizontal wave kinematics. Figs. 12 and 13 show the fluctuations in flapwise and edgewise Root Bending Moments (RBMs) as functions of wave phase and blade azimuth. These contours show a good qualitative agreement in the magnitude of blade RBM fluctuations in flapwise and edgewise directions between experiments and CFD, although still presenting the aforementioned underestimation in the amplitude of CFD load fluctuations.

The CFD results capture qualitatively well the flapwise RBM features, as shown in the figures, with a good agreement in blade phase and less so in lead with respect to wave phase. Peaks and troughs in the experimental RBMs lead wave elevation by approximately 25^∘^ and 45^∘^ in the f=0.40 Hz and f=0.65 Hz cases, respectively, while the CFD shows RBM peaks and troughs closer in phase with wave elevation but lagging wave peak by 30^∘^ to 40^∘^.

While the peak flapwise RBMs for the f=0.65 Hz wave are centred close to the top-dead centre (TDC, *ψ* ≈ 345^∘^), the f=0.40Hz case shows earlier peak flapwise loads that occur at blade phases *ψ* between 270^∘^ and 360^∘^, in a region of nearly horizontal blade positions in upstroke motion. Flapwise RBM minima are located, for both wave cases, at blade phases between 0^∘^ and 90^∘^ (i.e., between TDC and horizontal blade downstroke), with CFD minima being closer to the TDC than the experimental loads. It is noteworthy that blade flapwise RBMs show a much better agreement between experiments and CFD with respect to blade phase than with wave phase.

Similar to the flapwise RBMs, the edgewise RBMs in Fig. 13 show similar fluctuation amplitudes between experiments and CFD but differences in phase and banding patterns. The simulated edgewise RBMs show a more symmetrical behaviour in blade phase with respect to the TDC than the experimental results. The experiments show a well defined peak in blade phase close to the horizontal position in downstroke motion (*ψ* ≈ 90^∘^) which is not observed in the numerical results, while minima are centred around the TDC for both experiments and ALM. Wave phases exhibit the same differences previously observed in the flapwise RBMs, with peaks and troughs of edgewise RBMs leading wave elevation in the experiments and ALM results lagging the wave profile and being close to phase.

The underlying reason for the thrust and torque lead on wave elevation we observe in the experimental results is not well understood. The wave crest is aligned with the maximum horizontal component of wave-induced velocities across the water column, which, in the context of a quasi-steady analysis, could be intuitively associated with maximum loads. Actual phenomena are more complex, however, with added mass forces likely to play a role in phase shifting. McNaughton et al. (2025) theorised that in the case of vertical decay of wave kinematics, spanwise loading is better correlated when blades pass through the horizontal, leading to maximal and minimal loading at horizontal blade positions. The decorrelation of blade loads would likely lead to spanwise flows that, alongside other three-dimensional flow phenomena such as returning wake and wake perturbation effects (Smyth, 2020), could also alter the phase between loads and wave elevation.

**Fig. 14:**
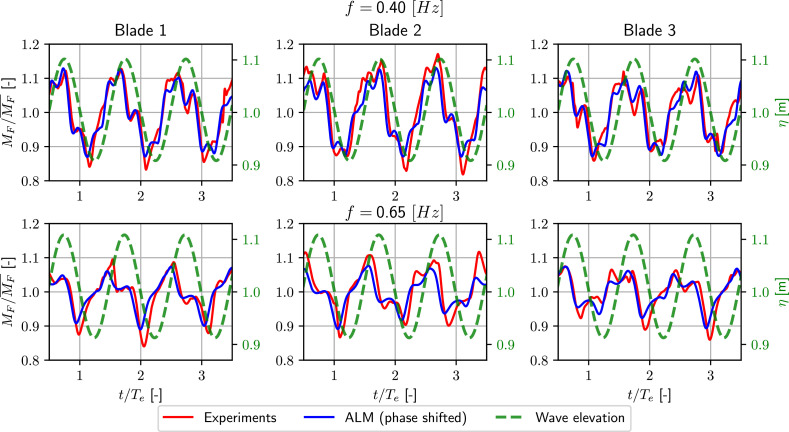
Flapwise root bending moments on the turbine blades measured from experiments and computed with the CFD-ALM simulations. The displayed time windows are selected as the best available match of wave and blade phases between experiments and phase shifted simulations.

**Fig. 15:**
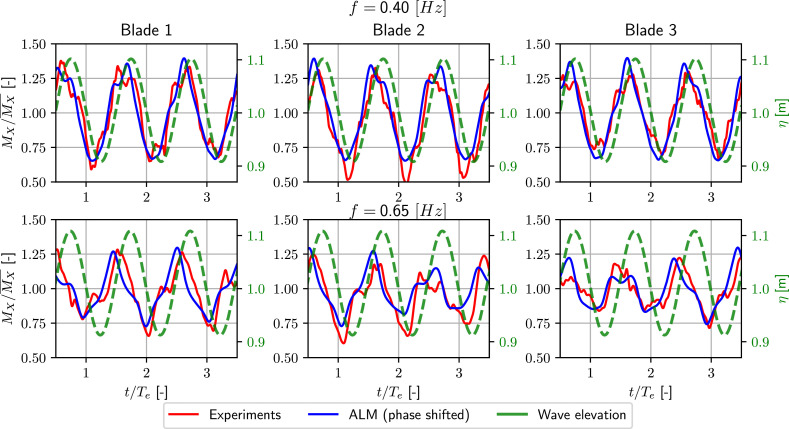
Edgewise root bending moments on the turbine blades measured from experiments and computed with the CFD-ALM simulations. The displayed time windows are selected as the best available match of wave and blade phases between experiments and phase shifted simulations.

From the different experimental results we observed a relatively consistent time lag between wave and thrust peaks across different frequencies, with a mean of 0.25 seconds and a standard deviation of 0.016 s. Applying this mean lag as a time shift to the numerical flapwise and edgewise RBMs and thus artificially matching blade and wave phase between the experimental and simulated results lead to the plots presented in Figs. 14 and 15. These plots compare modelled and experimental time series over three wave periods for two different wave frequencies for each of the three rotor blades.

The time series show the phase shifted ALM reproducing well the amplitudes of RBM fluctuations, as well as the general trends that are induced by the interplay between wave kinematics and blade encounter effects. As the rotor blades spin through the temporally- and spatially-varying flow field induced by wave kinematics, different blade sections sample through depth-dependent velocity amplitudes at different phases. This alters the inflow speed and leads to the multi harmonic patterns observed in the RBM plots. The results in the comparison show that the phase shifted ALM captures qualitatively well the general trends of peaks and troughs of RBM, especially in the flapwise direction, though with slightly lower amplitudes in the ALM results compared with experiments. This is in agreement with previous observations on integrated loads and phase RBM plots. The f=0.40Hz simulation shows the closest maxima and minima to experiments while the f=0.65Hz case shows a larger underestimation of RBMs. This, as discussed before, is at least partially associated with the numerical diffusion and underprediction of wave kinematics that increase with wave frequency. It is worth noting that the level of correlation in time shown in figures, however, was only seen using phase shifted results. This opens further questions about the physics captured by the ALM or for potential delays in the experimental data capture, though all streams of data were captured using the same data acquisition system.

Generally, both phase and time plots show a better agreement in flapwise than in edgewise RBMs. The uncertainty of edgewise RBMs is higher, with the assessment of torque-generating forces at blade level being challenging even experimentally. Flapwise loads are approximately an order of magnitude larger than the edgewise component, and are dominated by lift, while the loads in the edgewise direction are affected by both lift and drag forces; the latter being typically more challenging to compute accurately with numerical models and, thus, likely to exhibit higher error. Experimental edgewise results, on the other hand, rely on much more sensitive transducers than flapwise RBM measurements and require azimuthally-dependent corrections for blade weight and buoyancy, which are likely to increase uncertainty compared to flapwise measurements.

**Fig. 16:**
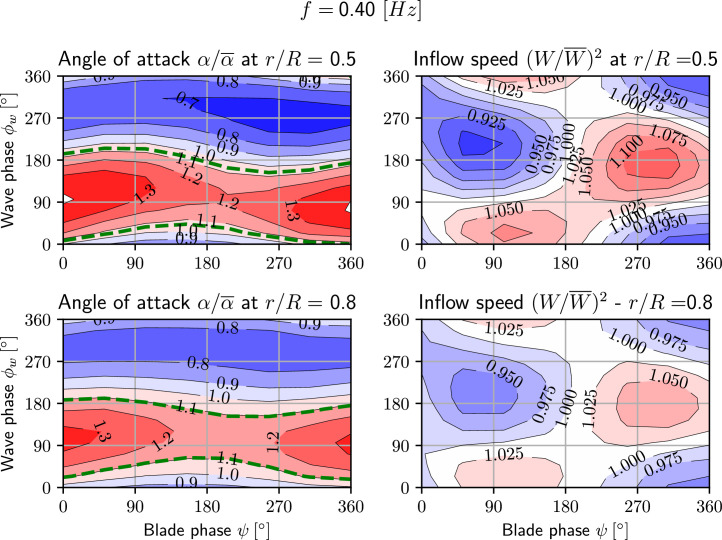
Relative changes in local angle of attack (*α*) and inflow speed relative to blade (*W*) squared at two radial locations for the f=0.40Hz case. Results are normalised by phase averaged angle of attack ($\bar{\alpha }$) and local inflow speed ($\bar{W}$), and presented as a function of wave (*ϕ_w_*) and blade (*ψ*) phases.

The patterns in the flapwise and edgewise RBM phase plots (Figs. 12 and 13) can be explained by the interactions of axial and vertical components of wave kinematics with the rotor blades. Fig. 16 shows contours of normalised angles of attack and inflow speed, at different blade and wave phases, for the longest wave case considered. The results are shown for two representative blade sections, and inflow speeds are squared to show relative impact on blade forces.

Plots of angle of attack show a pattern of horizontal bands that peak next to the TDC, with bands being approximately symmetrical around this blade phase due to stronger wave kinematics near the free surface. Peaks appear aligned or lagging slightly behind wave crest at *ϕ_w_* ≈ 90^∘^ which corresponds to the maximum wave-induced horizontal velocity. The angles of attack also show decreasing fluctuations as the tip is approached, which is consistent with a greater importance of the blades’ rotational speed in the velocity triangle further away from the rotor centre. Inflow speeds, on the contrary, show an anti-symmetric pattern to both blade and wave phases which differs from the angle of attack variation; maximum blade relative speeds occur at horizontal blade position when wave elevations are close to zero-crossing points. Fluctuations in inflow speed squared also decrease as the tip is approached, again due to the increasing preponderance of rotational speed.

**Fig. 17:**
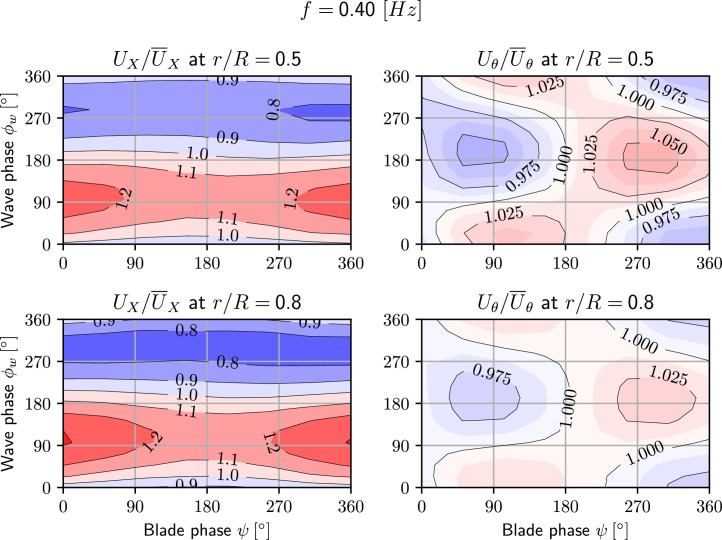
Relative changes in axial (*U_X_*) and tangential (*U_θ_*) speeds relative to blade at two radial locations for the f=0.40Hz case. Results are normalised by phase averaged axial ($\bar{U_{X}}$) and tangential local speeds ($\bar{U_{\theta }}$), and are presented as a function of wave (*ϕ_w_*) and blade (*ψ*) phases.

The axial and tangential speeds on the two analysed blade sections, presented in Fig. 17, show similar patterns to the angle of attack and inflow speed plots, respectively, indicating that changes in angle of attack are dominated by changes in axial flow speed, while the fluctuations on inflow speed are also associated with changes in relative speed in the tangent direction.

The axial and tangential velocity plots show the influence of the horizontal and vertical components of wave kinematics. In the analysed cases, with waves aligned with currents and no turbine yaw, the horizontal wave-induced velocities induce streamwise flow fluctuations and, thus, changes in angles of attack that affect the blade loads throughout the entire azimuth (although with a depth-dependent intensity). The vertical component of wave-induced velocity affects blades differently depending on blade azimuthal position. A horizontal blade during downstroke motion ($\psi =90^{\circ }$), e.g., will see a relative inflow speed reduction during the zero upcrossing at a wave phase of $\phi_{w}=180^{\circ }$ (i.e., maximum downwards wave-induced speed) but will increase its relative inflow speed during the upstroke motion ($\psi =270^{\circ }$) at the same wave phase. The effect, however, is non-linear and depends on the orientation of the blade sections, with maximum exposure to vertical kinematics at the horizontal ($\psi =90^{\circ }$ or $\psi =270^{\circ }$) and no effect, from the understanding of a two-dimensional blade-element model, at vertical positions ($\psi =0^{\circ }$ or $\psi =180^{\circ }$).

The combination of horizontal and vertical components of wave kinematics with blade azimuth is what ultimately drives the asymmetric patterns of peaks and troughs in the load fluctuations shown in Figs. 12 and 13. It does not fully explain, however, the azimuthal position of the peak loads, especially for the f=0.40Hz case where peak load fluctuations occur well before the blade reaches the TDC (*ψ* ≈ 300^∘^). Two further elements need to be considered: Fig. 16 marks with green dashed lines the limit at which lift coefficients abandon the linear range (*α* ≳ 8^∘^, as shown in Fig. 4). This implies that, even though substantial angle of attack fluctuations can be observed, the ALM does not linearly translate those to load changes beyond the green dashed line.

Constructive multi-rotor interference also affects thrust and power. It is of potentially lesser importance to dynamic loads than waves, but contributes towards the asymmetry observed in the RBM results. The increased resistance between rotors forces a larger mass-flux through the inter-rotor bypass, creating a flow acceleration zone which allows for higher angles of attack and, thus, higher loads when a blade passes next to that region (Mascrier et al., 2025, McNaughton et al., 2023). The direction of rotation and the position of the rotor in an array also affects the asymmetry in blade and wave phases seen for loads, angles of attack and relative inflow speeds. Changing the direction of rotation determines the blade phase at which positive or negative interactions between blade relative speed and vertical wave kinematics occur, while the relative position of the rotor in an array determines regions of bypass flow acceleration. The phase averaged results presented here, thus, are only representative of the left rotor in the work of McNaughton et al. (2025). While the general conclusions of the analysis presented in this paper hold for the opposite rotor, the different spatial configuration will lead to different RBM phase patterns which are discussed in the original reference (McNaughton et al., 2025).

The interactions between angles of attack and inflow speeds caused by waves and constructive interference, alongside blade characteristics and the onset of stall, drive load fluctuations which do not necessarily align with peak wave elevation or the TDC. As inflow conditions cross a blade-defined threshold, the thrust-producing capacity of the blade saturates, explaining the peak loads ahead of the TDC observed numerically and experimentally. The underlying cause for the phase between loads and wave elevation, however, is not clear and further investigation is required. We hypothesize, however, that wave-load phases could be either a consequence of phenomena not adequately captured by our numerical model (notably, due to the lack of an explicit treatment of added mass), or limitations in the measuring and/or data acquisition systems, the latter being less likely given that all sensors were connected to the same data acquisition system during the experimental campaign. The results shown by the ALM, however, are promising, with our work showing a good capacity to predict both steady and dynamic loads and power at rotor and blade level, both in terms of mean and fluctuating components, and at a computational cost far more manageable than blade resolved simulations.

### 3.3 Flow field analysis

**Fig. 18:**
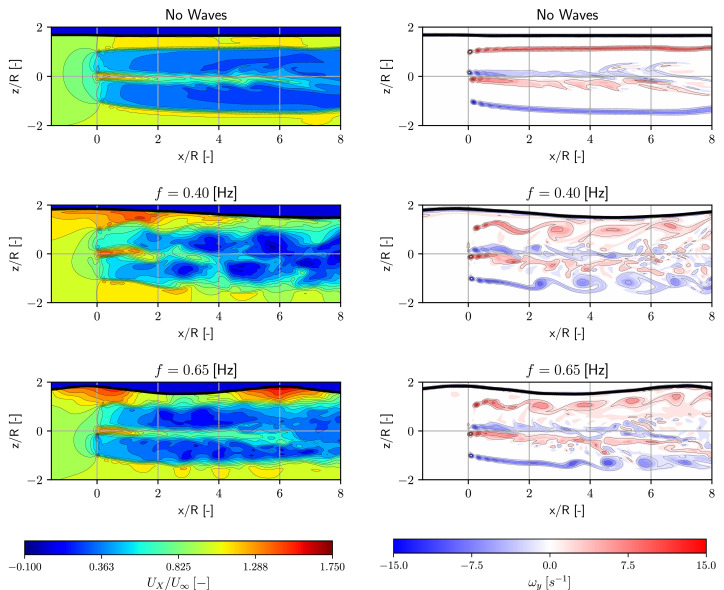
Instantaneous axial velocity and plane-normal vorticity contours *ω_y_*.

Fig. 18 shows instantaneous contours of axial velocity and plane-normal vorticity. The contours are plotted on vertical planes across the rotor centre, and display three of the analysed cases: No Waves, f=0.40 Hz and f=0.65Hz.

The three velocity contours show the wake behind the rotor with reduced speed, bypass zones between the wake and the free surface, and a strong jet through the centre of the rotor due to the lack of a nacelle in the model. The case without waves shows a stable wake up to *x*/*R* ≈ 5.0 and minor instabilities initiating from shear layer rollup thereafter, while the cases with waves show strong perturbations of the wake and waves triggering instabilities in the near wake region, increasing the mixing between the wake and outer flows.

The horizontal and vertical components of wave kinematics affect the wake differently. Horizontal wave kinematics act on the tip vortices squeezing and stretching them periodically, facilitating vortex merging as observed in the vorticity contours in Fig. 18, and triggering the formation of larger vortical structures, as seen in the vorticity contours where *x*/*R* > 2.0 in the cases with waves.

Vertical wave kinematics affect the wake by fostering momentum exchange with the outer flow. Vertical wave-induced velocities transport the vorticity sheet that shields the wake vertically, seen in the vorticity contours without waves, creating vertical perturbations that are periodic in space and time, and providing increased oscillation to the large coherent structures that arise from the merging of the tip vortices. The perturbation of the wake’s vorticity sheet to faster flows in the bypass increase momentum exchange by either exposing part of the slow wake to the faster bypass flow, or by entraining part of the outer flow into the wake.

The merging of tip vortices, alongside the momentum exchange and flow rotation along the edge of the wake, trigger and support the formation of large three-dimensional vortical structures that travel along the domain. Part of the formation process can be seen in the vorticity contours with waves, Fig. 18, in the near wake region where *x*/*R* < 2.0 and is especially apparent in proximity to the free surface. The process of vortex merging is also evident in the three dimensional isosurfaces of Q-contour in Fig. 20 for the three cases with waves that are displayed. The Q-contours also show that interactions between waves, wake and tip vortices result in large three-dimensional structures, which are observed to take either helical or vortex ring patterns, and become much larger in size and travel further than the original tip vortices seen in the case without waves for the set level of Q.

**Fig. 19:**
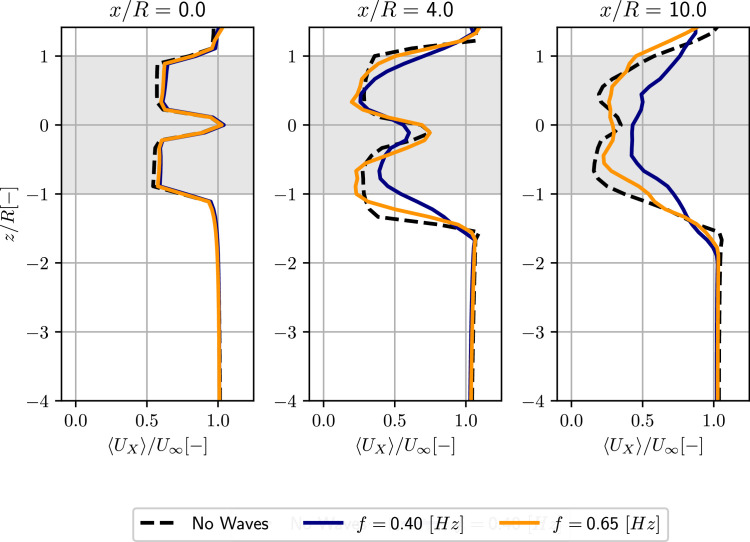
Time-averaged profiles of axial velocity across the water column ⟨*U_X_*⟩ at different streamwise locations.

**Fig. 20:**
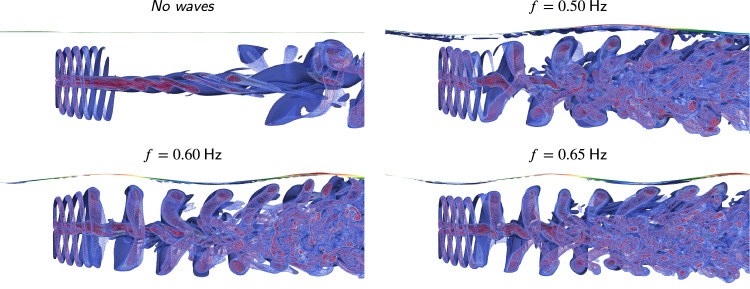
Instantaneous contours of Q-criterion for four different wave conditions: no wave, and wave frequencies of 0.50 Hz, 0.60 Hz, and 0.65 Hz. The flow is from left to right.

The increased momentum exchange between wake and outer flow leads to a faster recovery of kinetic energy in the wake region. Fig. 19 shows profiles of time-averaged axial velocity and compares cases with and without waves. From these results we observe that no significant differences exist in mean flow speed at the rotor plane between cases. However, the near and far wake profiles show faster wake recovery due to wave-induced mixing, with longer waves producing stronger flow fluctuations, mixing, and, thus, faster wake recovery and flow speeds downstream from the rotor plane. The x/R=10 section, in particular, shows that the f=0.65 Hz and f=0.40 Hz wave cases have vertically averaged velocities across the rotor projected area that are 22% and 104% faster than the case without waves, which translates, respectively, to an approximate of 1.8 to 8.5 fold increase in mean kinetic energy flux at this location.

The rotor alters the flow field and induces speed gradients across the domain. The wake region is the most evident area where the rotor causes a strong velocity deficit, but in addition and as shown in Fig. 18, the rotor has a significant impact slowing the flow upstream and creating high-speed bypass zones. To evaluate the impact of rotor-induced velocity gradients and how they can affect wave propagation across the domain, we evaluated the phase *ϕ_K_* between undisturbed and rotor-affected axial velocity fluctuations, with the undisturbed case corresponding to horizontal wave kinematics on top of current speed. Note that positive *ϕ_K_* angles indicate a phase-lead in rotor-perturbed cases.

**Fig. 21:**
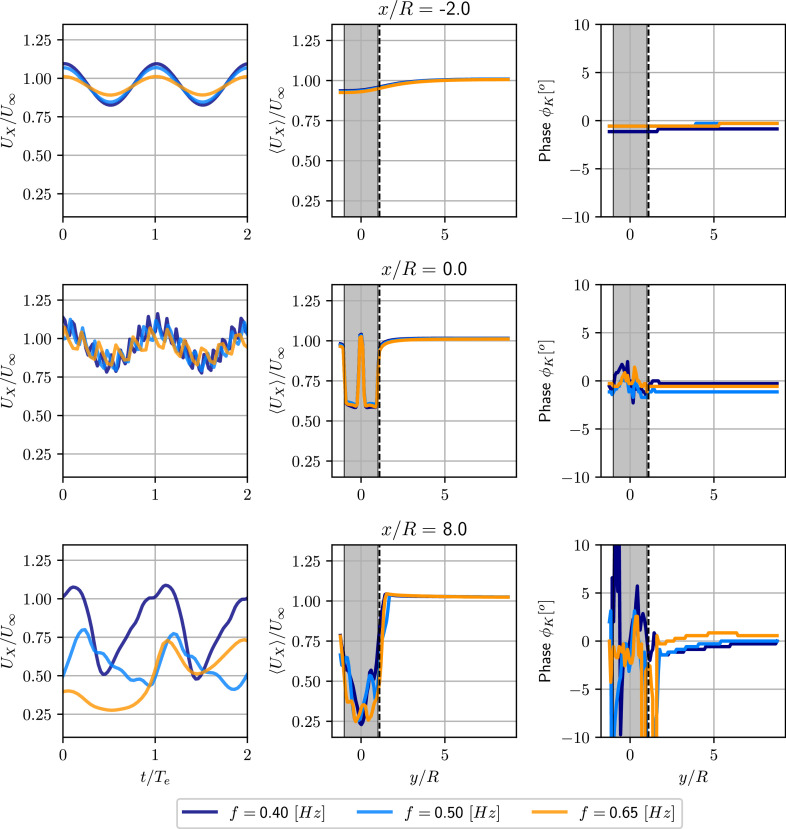
Evaluation of the flow field at different streamwise locations across horizontal planes at hub-height, z/R=0. The first, second and third columns show, respectively, streamwise velocity time series at y/R=1.1, i.e., just outside of the rotor swept area (marked with a dashed line on the second and third column plots); time averaged flow speed; and phase angle of the streamwise kinematics relative to undisturbed flow kinematics.

The phase results were evaluated on a horizontal plane at hub-height z/R=0 and are presented in Fig. 21. The figure shows three columns: time-series of axial speed at a fixed lateral position, time-averaged velocity profiles, and phases *ϕ_K_*. The time series serve as an example of the processed data, and are extracted at a lateral position just outside of the rotor swept area at y/R=1.1. The three rows show data extracted at three axial locations: upstream, x/R=−2.0; rotor plane, x/R=0.0; and downstream, x/R=8.0.

The upstream plots in Fig. 21 show harmonic velocity fluctuations free from significant influences from sources other than waves, time-averaged flow speeds which show a limited impact of rotor induction, and negligible changes in phase for all wave cases. The rotor plane section shows time series which are dominated by harmonics at the wave frequency, but with secondary high-frequency constituents associated with blade passing. Small perturbations to wave phase at the rotor region and smaller phase changes in the bypass are observed (*ϕ_K_* < 1^∘^), but a clear trend across wave frequencies cannot be identified. Finally, the time series downstream show the impact of an expanding streamtube, as the sampling point falls inside the wake. In this case, the identification of harmonics and phases become significantly more challenging, as shown by the time-series and phase plots. Outside the wake region, we observe some minor changes in phase angle with the longer and shorter waves showing the largest and smallest phase angles, respectively.

The results show typical lag phases across the rotor wake and bypass less than 1^∘^, indicating a limited impact of rotor induction on the phase angle of wave kinematics. This is likely the consequence of rotor induced velocities being considerably smaller than the wave propagation speeds (the waves in this study range between 3.4 m/s ≤ (*L*/*T_e_*) ≤ 4.9 m/s), as well as to zones of rotor-perturbed flow speed spanning only a limited portion of the domain. The small phase angles *ϕ_K_* also indicate that the phase lead of root bending moments over wave elevation, discussed in Section 3.2, is not likely a consequence of the rotor perturbing the wave field. We conclude that wave phase angles do not divert significantly from undisturbed waves throughout the domain except for the wake region, where the wave profile and thus kinematics are altered by the rotor upstream.

## 4 Conclusions

Through the comparisons with experiments, this study demonstrated the capacity of the presented Actuator Line Model (ALM) to accurately reproduce the steady and dynamic loads and power fluctuations on a tidal turbine operating in proximity to the free surface and in the presence of surface waves over the range of analysed frequencies. The method accuracy and robustness, however, are not granted and rely on submodel implementation and underlying assumptions (Willden et al., 2023). These include the size and shape of the force projection in the fluid domain, the sampling method, and the polar coefficients.

The sampling method of Zormpa et al. (2025) was tested under steady and dynamic inflow conditions, while the use of steady lift and drag coefficients extracted from blade resolved simulations permits the model to accurately capture spanwise load variations and tip-loss effects through radially-varying polar coefficients (Wimshurst and Willden, 2018). These two aspects enabled our ALM to correctly predict steady thrust and power with relative differences below 1% at design tip-speed ratio.

The comparisons between our model and the experiments under a deterministic dynamic inflow driven by surface waves is another novel element in this work. The ALM predictions of the rotor performance in waves show a similar level of agreement to the steady-state cases in terms of time-averaged integrated thrust and power, and a good qualitative agreement on dynamic loads and power. Standard deviations of the dynamic load time series show the CFD model underestimating power and thrust fluctuations in range of 3.7%-15.3% and 13.2%-28.9%, respectively, for wave frequencies between 0.4 and 0.65 Hz. Discrepancies are mainly attributed to wave modelling errors through numerical wave dissipation that lead to lower amplitude wave kinematics at the rotor plane, alongside potential limitations in the ALM, which may include the use of steady-state polar coefficients and the lack of an explicit treatment for inertial forces.

The unrestricted access to the rotor load calculations and the simulated flow field in the numerical model allows to reassess the previous experimental campaign, using the numerical tool to improve physical understanding. Single-blade load fluctuations show a good agreement between model and experiments, particularly in terms of maxima and minima, as well as in blade phase distributions. The peak loads at blade phase near the horizontal and during upstroke motion, observed in both experiments and simulations, were explained by the influence of the two components of wave kinematics, horizontal and vertical, and their impact on sectional inflow speed and angle of attack. The latter, in particular, was shown to exceed the linear range of lift coefficients when crossing the bypass region over sections of the blade where most thrust and power are produced. This leads to the saturation of the thrust-generating capacity of the blades and, thus, peak loads occurring ahead of the top-dead centre, confirming previous speculations presented in McNaughton et al. (2025).

Flapwise and edgewise root bending moment distributions in wave phase showed a significant offset between model and experiments, up to ca. 45^∘^, with loads leading wave elevation in the experiments and the ALM lagging but closer to phase. We speculate this could be a consequence of not adequately modelling inertial loads, added-mass and Froude-Krylov components. The similar time lag between wave elevation and peak loads at different frequencies in the experimental results of approximately a quarter-second, as well as the good agreement observed in time series between CFD and experiments after matching blade and time-shifted wave phases, suggest that phase differences could also be a consequence of a data acquisition delay. This, however, appears unlikely considering that wave gauges and force transducers were connected and synchronised using a single data acquisition system.

Inertial forces have been implemented in blade-element theory based models by adding an inertial term similar to that used in Morison’s equation (Morison et al., 1950) treating the foil sections as flat plates. Such an approach, however, shows a limited impact on dynamic loads (Faudot and Dahlhaug, 2012, Maniaci and Li, 2012, Murray et al., 2018). Thus, while the introduction of explicit terms for added-mass can be straightforward, we consider that necessary improvements to the ALM prediction of the phase of unsteady blade loading relative to wave elevation should rely on further investigations of the physical mechanisms that impact wave-induced inertial forces on tidal turbines blades over different wave frequencies and amplitudes. Further experiments are thus required to develop a complete understanding of the phenomena driving unsteady loading on rotor blades and the phasing between loads and wave elevation.

The flow analysis on the CFD simulations showed a substantial impact of waves on wake recovery. We observed a substantial increase in the time-averaged axial flow speed in the wake in presence of waves, compared to the case without, as the far wake region is entered. This suggests that waves could be beneficial to tidal farm energy yields as well as showing a potential for further farm layout optimisation. The presence of waves, however, not only affects dynamic loads directly, but also triggers wake structure merger and the formation of large coherent structures that are likely to have further impact on the dynamics and fatigue loads of rotors downstream. Finally, while turbine induction causes a lag in the phase of wave propagation, the impact is limited and typically below 1 ^∘^ phase for the analysed cases.

## CRediT authorship contribution statement

**Federico Zilic de Arcos:** Writing – original draft, Visualization, Validation, Supervision, Software, Resources, Project administration, Methodology, Investigation, Funding acquisition, Formal analysis, Data curation, Conceptualization. **James McNaughton:** Writing – review & editing, Methodology, Investigation, Formal analysis, Data curation. **Christopher R. Vogel:** Writing – review & editing, Methodology, Conceptualization. **Richard H․ J. Willden:** Writing – review & editing, Resources, Funding acquisition, Formal analysis. **Grégory Pinon:** Writing – review & editing, Supervision, Resources, Project administration, Investigation, Funding acquisition, Formal analysis.

## Declaration of competing interest

The authors declare that they have no known competing financial interests or personal relationships that could have appeared to influence the work reported in this paper.
